# Supplementary material for: Pyruvate Dehydrogenase Kinase 1 inhibition mediated oxidative phosphorylation enhancement in cartilage promotes osteoarthritis progression
Source: BMC Musculoskelet Disord. 2023 Jul 20;24:597. doi: 10.1186/s12891-023-06585-6 (PMC10357736; doi:10.1186/s12891-023-06585-6)
Supplement: Supplementary file 1 — Additional file 1. [file 12891_2023_6585_MOESM1_ESM.zip › KEGG.Gsea.1653622432464/KEGG_FC_GAMMA_R_MEDIATED_PHAGOCYTOSIS.html]

Details for gene set KEGG\_FC\_GAMMA\_R\_MEDIATED\_PHAGOCYTOSIS[GSEA]

|  || Dataset | 55457\_collapsed\_to\_symbols.cls.cls #OA\_versus\_normal.cls.cls #OA\_versus\_normal\_repos |
| Phenotype | cls.cls#OA\_versus\_normal\_repos |
| Upregulated in class | normal |
| GeneSet | KEGG\_FC\_GAMMA\_R\_MEDIATED\_PHAGOCYTOSIS |
| Enrichment Score (ES) | -0.19544253 |
| Normalized Enrichment Score (NES) | -0.8354916 |
| Nominal p-value | 0.80546623 |
| FDR q-value | 0.8997981 |
| FWER p-Value | 1.0 |
Table: GSEA Results Summary

  

Fig 1: Enrichment plot: KEGG\_FC\_GAMMA\_R\_MEDIATED\_PHAGOCYTOSIS      
 Profile of the Running ES Score & Positions of GeneSet Members on the Rank Ordered List

  

| SYMBOL | TITLE | RANK IN GENE LIST | RANK METRIC SCORE | RUNNING ES | CORE ENRICHMENT || 1 | ARPC1A | actin related protein 2/3 complex subunit 1A [Source:HGNC Symbol;Acc:HGNC:703] | 47 | 1.003 | 0.0315 | No |
| 2 | DOCK2 | dedicator of cytokinesis 2 [Source:HGNC Symbol;Acc:HGNC:2988] | 520 | 0.652 | 0.0176 | No |
| 3 | VAV1 | vav guanine nucleotide exchange factor 1 [Source:HGNC Symbol;Acc:HGNC:12657] | 601 | 0.622 | 0.0332 | No |
| 4 | FCGR1A | Fc fragment of IgG receptor Ia [Source:HGNC Symbol;Acc:HGNC:3613] | 633 | 0.610 | 0.0522 | No |
| 5 | PLCG2 | phospholipase C gamma 2 [Source:HGNC Symbol;Acc:HGNC:9066] | 725 | 0.588 | 0.0657 | No |
| 6 | MAPK3 | mitogen-activated protein kinase 3 [Source:HGNC Symbol;Acc:HGNC:6877] | 757 | 0.579 | 0.0836 | No |
| 7 | PLPP1 | phospholipid phosphatase 1 [Source:HGNC Symbol;Acc:HGNC:9228] | 1109 | 0.494 | 0.0736 | No |
| 8 | ARPC5 | actin related protein 2/3 complex subunit 5 [Source:HGNC Symbol;Acc:HGNC:708] | 1118 | 0.492 | 0.0902 | No |
| 9 | AKT1 | AKT serine/threonine kinase 1 [Source:HGNC Symbol;Acc:HGNC:391] | 1192 | 0.478 | 0.1013 | No |
| 10 | PIP5K1B | phosphatidylinositol-4-phosphate 5-kinase type 1 beta [Source:HGNC Symbol;Acc:HGNC:8995] | 1291 | 0.459 | 0.1098 | No |
| 11 | FCGR2C | Fc fragment of IgG receptor IIc (gene/pseudogene) [Source:HGNC Symbol;Acc:HGNC:15626] | 1460 | 0.435 | 0.1119 | No |
| 12 | ASAP3 | "ArfGAP with SH3 domain, ankyrin repeat and PH domain 3 [Source:HGNC Symbol;Acc:HGNC:14987]" | 1480 | 0.432 | 0.1256 | No |
| 13 | ARPC3 | actin related protein 2/3 complex subunit 3 [Source:HGNC Symbol;Acc:HGNC:706] | 1743 | 0.393 | 0.1189 | No |
| 14 | FCGR2B | Fc fragment of IgG receptor IIb [Source:HGNC Symbol;Acc:HGNC:3618] | 2016 | 0.360 | 0.1103 | No |
| 15 | CRK | "CRK proto-oncogene, adaptor protein [Source:HGNC Symbol;Acc:HGNC:2362]" | 2018 | 0.360 | 0.1229 | No |
| 16 | ARPC1B | actin related protein 2/3 complex subunit 1B [Source:HGNC Symbol;Acc:HGNC:704] | 2184 | 0.340 | 0.1219 | No |
| 17 | PIK3CB | "phosphatidylinositol-4,5-bisphosphate 3-kinase catalytic subunit beta [Source:HGNC Symbol;Acc:HGNC:8976]" | 2186 | 0.340 | 0.1338 | No |
| 18 | NCF1 | neutrophil cytosolic factor 1 [Source:HGNC Symbol;Acc:HGNC:7660] | 2195 | 0.339 | 0.1450 | No |
| 19 | PRKCD | protein kinase C delta [Source:HGNC Symbol;Acc:HGNC:9399] | 2237 | 0.335 | 0.1536 | No |
| 20 | CRKL | "CRK like proto-oncogene, adaptor protein [Source:HGNC Symbol;Acc:HGNC:2363]" | 2283 | 0.329 | 0.1616 | No |
| 21 | SPHK2 | sphingosine kinase 2 [Source:HGNC Symbol;Acc:HGNC:18859] | 2478 | 0.305 | 0.1572 | No |
| 22 | PLA2G4A | phospholipase A2 group IVA [Source:HGNC Symbol;Acc:HGNC:9035] | 2656 | 0.284 | 0.1533 | No |
| 23 | ARPC2 | actin related protein 2/3 complex subunit 2 [Source:HGNC Symbol;Acc:HGNC:705] | 2893 | 0.261 | 0.1441 | No |
| 24 | INPP5D | inositol polyphosphate-5-phosphatase D [Source:HGNC Symbol;Acc:HGNC:6079] | 3085 | 0.241 | 0.1377 | No |
| 25 | WASF2 | WASP family member 2 [Source:HGNC Symbol;Acc:HGNC:12733] | 3101 | 0.239 | 0.1449 | No |
| 26 | PIP4K2B | phosphatidylinositol-5-phosphate 4-kinase type 2 beta [Source:HGNC Symbol;Acc:HGNC:8998] | 3416 | 0.210 | 0.1277 | No |
| 27 | MARCKS | myristoylated alanine rich protein kinase C substrate [Source:HGNC Symbol;Acc:HGNC:6759] | 3653 | 0.190 | 0.1160 | No |
| 28 | PRKCA | protein kinase C alpha [Source:HGNC Symbol;Acc:HGNC:9393] | 3728 | 0.183 | 0.1166 | No |
| 29 | PAK1 | p21 (RAC1) activated kinase 1 [Source:HGNC Symbol;Acc:HGNC:8590] | 3861 | 0.169 | 0.1123 | No |
| 30 | LAT | linker for activation of T cells [Source:HGNC Symbol;Acc:HGNC:18874] | 3922 | 0.162 | 0.1133 | No |
| 31 | VAV3 | vav guanine nucleotide exchange factor 3 [Source:HGNC Symbol;Acc:HGNC:12659] | 4082 | 0.148 | 0.1060 | No |
| 32 | SYK | spleen associated tyrosine kinase [Source:HGNC Symbol;Acc:HGNC:11491] | 4084 | 0.147 | 0.1111 | No |
| 33 | FCGR3A | Fc fragment of IgG receptor IIIa [Source:HGNC Symbol;Acc:HGNC:3619] | 4594 | 0.105 | 0.0751 | No |
| 34 | VASP | vasodilator stimulated phosphoprotein [Source:HGNC Symbol;Acc:HGNC:12652] | 5143 | 0.060 | 0.0344 | No |
| 35 | RPS6KB2 | ribosomal protein S6 kinase B2 [Source:HGNC Symbol;Acc:HGNC:10437] | 5496 | 0.033 | 0.0081 | No |
| 36 | ASAP2 | "ArfGAP with SH3 domain, ankyrin repeat and PH domain 2 [Source:HGNC Symbol;Acc:HGNC:2721]" | 5742 | 0.012 | -0.0106 | No |
| 37 | PRKCE | protein kinase C epsilon [Source:HGNC Symbol;Acc:HGNC:9401] | 5926 | 0.000 | -0.0249 | No |
| 38 | DNM3 | dynamin 3 [Source:HGNC Symbol;Acc:HGNC:29125] | 6020 | -0.006 | -0.0319 | No |
| 39 | RAC2 | Rac family small GTPase 2 [Source:HGNC Symbol;Acc:HGNC:9802] | 6098 | -0.013 | -0.0375 | No |
| 40 | RAF1 | "Raf-1 proto-oncogene, serine/threonine kinase [Source:HGNC Symbol;Acc:HGNC:9829]" | 6187 | -0.019 | -0.0437 | No |
| 41 | FCGR2A | Fc fragment of IgG receptor IIa [Source:HGNC Symbol;Acc:HGNC:3616] | 6329 | -0.031 | -0.0536 | No |
| 42 | PRKCB | protein kinase C beta [Source:HGNC Symbol;Acc:HGNC:9395] | 6453 | -0.039 | -0.0619 | No |
| 43 | LYN | "LYN proto-oncogene, Src family tyrosine kinase [Source:HGNC Symbol;Acc:HGNC:6735]" | 6514 | -0.043 | -0.0650 | No |
| 44 | PIP5K1C | phosphatidylinositol-4-phosphate 5-kinase type 1 gamma [Source:HGNC Symbol;Acc:HGNC:8996] | 6601 | -0.050 | -0.0700 | No |
| 45 | WAS | WASP actin nucleation promoting factor [Source:HGNC Symbol;Acc:HGNC:12731] | 6666 | -0.054 | -0.0731 | No |
| 46 | DNM1 | dynamin 1 [Source:HGNC Symbol;Acc:HGNC:2972] | 6691 | -0.055 | -0.0730 | No |
| 47 | HCK | "HCK proto-oncogene, Src family tyrosine kinase [Source:HGNC Symbol;Acc:HGNC:4840]" | 6788 | -0.062 | -0.0784 | No |
| 48 | MAPK1 | mitogen-activated protein kinase 1 [Source:HGNC Symbol;Acc:HGNC:6871] | 6853 | -0.068 | -0.0810 | No |
| 49 | ARPC5L | actin related protein 2/3 complex subunit 5 like [Source:HGNC Symbol;Acc:HGNC:23366] | 7023 | -0.082 | -0.0913 | No |
| 50 | RAC1 | Rac family small GTPase 1 [Source:HGNC Symbol;Acc:HGNC:9801] | 7192 | -0.096 | -0.1011 | No |
| 51 | PIK3CD | "phosphatidylinositol-4,5-bisphosphate 3-kinase catalytic subunit delta [Source:HGNC Symbol;Acc:HGNC:8977]" | 7405 | -0.111 | -0.1137 | No |
| 52 | MARCKSL1 | MARCKS like 1 [Source:HGNC Symbol;Acc:HGNC:7142] | 7621 | -0.129 | -0.1260 | No |
| 53 | PIK3CA | "phosphatidylinositol-4,5-bisphosphate 3-kinase catalytic subunit alpha [Source:HGNC Symbol;Acc:HGNC:8975]" | 7733 | -0.136 | -0.1299 | No |
| 54 | GAB2 | GRB2 associated binding protein 2 [Source:HGNC Symbol;Acc:HGNC:14458] | 7742 | -0.137 | -0.1257 | No |
| 55 | PTPRC | protein tyrosine phosphatase receptor type C [Source:HGNC Symbol;Acc:HGNC:9666] | 7744 | -0.137 | -0.1209 | No |
| 56 | PIK3CG | "phosphatidylinositol-4,5-bisphosphate 3-kinase catalytic subunit gamma [Source:HGNC Symbol;Acc:HGNC:8978]" | 7953 | -0.152 | -0.1319 | No |
| 57 | AKT3 | AKT serine/threonine kinase 3 [Source:HGNC Symbol;Acc:HGNC:393] | 8008 | -0.156 | -0.1306 | No |
| 58 | DNM1L | dynamin 1 like [Source:HGNC Symbol;Acc:HGNC:2973] | 8499 | -0.194 | -0.1621 | No |
| 59 | GSN | gelsolin [Source:HGNC Symbol;Acc:HGNC:4620] | 8507 | -0.194 | -0.1558 | No |
| 60 | PRKCG | protein kinase C gamma [Source:HGNC Symbol;Acc:HGNC:9402] | 8582 | -0.200 | -0.1545 | No |
| 61 | PIK3R3 | phosphoinositide-3-kinase regulatory subunit 3 [Source:HGNC Symbol;Acc:HGNC:8981] | 8801 | -0.217 | -0.1640 | No |
| 62 | PIK3R5 | phosphoinositide-3-kinase regulatory subunit 5 [Source:HGNC Symbol;Acc:HGNC:30035] | 8804 | -0.217 | -0.1565 | No |
| 63 | MYO10 | myosin X [Source:HGNC Symbol;Acc:HGNC:7593] | 8825 | -0.219 | -0.1503 | No |
| 64 | PLD1 | phospholipase D1 [Source:HGNC Symbol;Acc:HGNC:9067] | 9365 | -0.261 | -0.1833 | No |
| 65 | ARPC4 | actin related protein 2/3 complex subunit 4 [Source:HGNC Symbol;Acc:HGNC:707] | 9522 | -0.276 | -0.1858 | Yes |
| 66 | PLD2 | phospholipase D2 [Source:HGNC Symbol;Acc:HGNC:9068] | 9615 | -0.284 | -0.1830 | Yes |
| 67 | SCIN | scinderin [Source:HGNC Symbol;Acc:HGNC:21695] | 9742 | -0.296 | -0.1824 | Yes |
| 68 | LIMK1 | LIM domain kinase 1 [Source:HGNC Symbol;Acc:HGNC:6613] | 9746 | -0.296 | -0.1723 | Yes |
| 69 | LIMK2 | LIM domain kinase 2 [Source:HGNC Symbol;Acc:HGNC:6614] | 9812 | -0.302 | -0.1667 | Yes |
| 70 | PIP5K1A | phosphatidylinositol-4-phosphate 5-kinase type 1 alpha [Source:HGNC Symbol;Acc:HGNC:8994] | 9851 | -0.306 | -0.1589 | Yes |
| 71 | PLPP3 | phospholipid phosphatase 3 [Source:HGNC Symbol;Acc:HGNC:9229] | 9906 | -0.312 | -0.1522 | Yes |
| 72 | AMPH | amphiphysin [Source:HGNC Symbol;Acc:HGNC:471] | 10043 | -0.326 | -0.1514 | Yes |
| 73 | MAP2K1 | mitogen-activated protein kinase kinase 1 [Source:HGNC Symbol;Acc:HGNC:6840] | 10197 | -0.340 | -0.1514 | Yes |
| 74 | RPS6KB1 | ribosomal protein S6 kinase B1 [Source:HGNC Symbol;Acc:HGNC:10436] | 10224 | -0.343 | -0.1413 | Yes |
| 75 | ASAP1 | "ArfGAP with SH3 domain, ankyrin repeat and PH domain 1 [Source:HGNC Symbol;Acc:HGNC:2720]" | 10276 | -0.349 | -0.1331 | Yes |
| 76 | WASF3 | WASP family member 3 [Source:HGNC Symbol;Acc:HGNC:12734] | 10316 | -0.352 | -0.1238 | Yes |
| 77 | PLCG1 | phospholipase C gamma 1 [Source:HGNC Symbol;Acc:HGNC:9065] | 10722 | -0.395 | -0.1415 | Yes |
| 78 | PIK3R2 | phosphoinositide-3-kinase regulatory subunit 2 [Source:HGNC Symbol;Acc:HGNC:8980] | 10956 | -0.425 | -0.1448 | Yes |
| 79 | ARF6 | ADP ribosylation factor 6 [Source:HGNC Symbol;Acc:HGNC:659] | 11084 | -0.442 | -0.1392 | Yes |
| 80 | PIK3R1 | phosphoinositide-3-kinase regulatory subunit 1 [Source:HGNC Symbol;Acc:HGNC:8979] | 11099 | -0.443 | -0.1247 | Yes |
| 81 | PLA2G6 | phospholipase A2 group VI [Source:HGNC Symbol;Acc:HGNC:9039] | 11128 | -0.446 | -0.1112 | Yes |
| 82 | PLPP2 | phospholipid phosphatase 2 [Source:HGNC Symbol;Acc:HGNC:9230] | 11160 | -0.452 | -0.0978 | Yes |
| 83 | WASF1 | WASP family member 1 [Source:HGNC Symbol;Acc:HGNC:12732] | 11387 | -0.484 | -0.0984 | Yes |
| 84 | VAV2 | vav guanine nucleotide exchange factor 2 [Source:HGNC Symbol;Acc:HGNC:12658] | 11413 | -0.489 | -0.0832 | Yes |
| 85 | WASL | WASP like actin nucleation promoting factor [Source:HGNC Symbol;Acc:HGNC:12735] | 12207 | -0.647 | -0.1224 | Yes |
| 86 | SPHK1 | sphingosine kinase 1 [Source:HGNC Symbol;Acc:HGNC:11240] | 12320 | -0.682 | -0.1072 | Yes |
| 87 | PIKFYVE | "phosphoinositide kinase, FYVE-type zinc finger containing [Source:HGNC Symbol;Acc:HGNC:23785]" | 12347 | -0.690 | -0.0850 | Yes |
| 88 | CFL1 | cofilin 1 [Source:HGNC Symbol;Acc:HGNC:1874] | 12352 | -0.691 | -0.0611 | Yes |
| 89 | DNM2 | dynamin 2 [Source:HGNC Symbol;Acc:HGNC:2974] | 12362 | -0.695 | -0.0374 | Yes |
| 90 | CDC42 | cell division cycle 42 [Source:HGNC Symbol;Acc:HGNC:1736] | 12681 | -0.852 | -0.0323 | Yes |
| 91 | AKT2 | AKT serine/threonine kinase 2 [Source:HGNC Symbol;Acc:HGNC:392] | 12896 | -1.404 | 0.0003 | Yes |
Table: GSEA details [plain text format]

  

Fig 2: KEGG\_FC\_GAMMA\_R\_MEDIATED\_PHAGOCYTOSIS      
 Blue-Pink O' Gram in the Space of the Analyzed GeneSet

  

Fig 3: KEGG\_FC\_GAMMA\_R\_MEDIATED\_PHAGOCYTOSIS: Random ES distribution      
 Gene set null distribution of ES for **KEGG\_FC\_GAMMA\_R\_MEDIATED\_PHAGOCYTOSIS**
